# Bifunctional Mesoporous Carbon Nitride: Highly Efficient Enzyme-like Catalyst for One-pot Deacetalization-Knoevenagel Reaction

**DOI:** 10.1038/srep12901

**Published:** 2015-08-05

**Authors:** Lin Zhong, Chokkalingam Anand, Kripal Singh Lakhi, Geoffrey Lawrence, Ajayan Vinu

**Affiliations:** 1 Australian Institute for Bioengineering and Nanotechnology, University of Queensland, Brisbane, Queensland 4072, Australia; 2 College of Chemical Engineering, Sichuan University, Chengdu, Sichuan 610065, China

## Abstract

Recently, mesoporous carbon nitride (MCN) has aroused extensive interest for its potential applications in organocatalysis, photo- and electrochemistry and CO_2_ capture. However, further surface functionalization of MCN for advanced nanomaterials and catalysis still remains very challenging. Here we show that acidic carboxyl groups can be smoothly introduced onto the surface of well-ordered MCN without annihilation between the introduced acid groups and MCN’s inherent basic groups through a facile UV light oxidation method. The functionalization generates a novel bifunctional nanocatalyst which offers an enzyme-like catalytic performance in the one-pot deacetalization-Knoevenagel reaction of benzaldehyde dimethylacetal and malononitrile with 100% conversion and more than 99% selectivity due to the cooperative catalysis between the acid and base groups separated on the surface of the catalyst. The results provide a general method to create multifunctional nanomaterials and open new opportunities for the development of high efficient catalyst for green organic synthesis.

Organic synthesis has a significant impact on the development of modern civilization. However, the major drawbacks associated with organic synthesis processes are that they are energetically intensive and cause severe environmental degradation. Synthetic chemists have addressed these drawbacks by developing economical and environmentally benign synthetic processes^1^,^2^. Although there have been great achievements in organic synthesis recent years, scientists still have a long way to realize the goal of “perfect chemical reactions” with 100% yields and 100% selectivity^3^. However, it seems that nature can provide abundant and extraordinary paradigms for these efficient processes, where diversiform enzymes with two or more catalytic active sites cooperate synergistically or in a designated sequence for a specific transformation with a high activity and selectivity but without requisition of intermediates separation^4^,^5^. Inspired by the elegance of enzymatic catalysis, one-pot catalytic organic syntheses in which multiple catalytic reactions are combined into one synthetic operation are currently receiving considerable attention and have been intensively reported^6^,^7^,^8^,^9^. However, the design of multifunctional catalyst systems for one-pot reactions remains very challenging due to incompatibility of active sites, leaching of metal ions, and difficulty in recovering of catalysts^10^,^11^. “Anchoring” and separating multiple catalytic active sites, especially acidic and basic sites on solid without any metal may provide opportunities for developing stable enzyme-like heterogeneous catalysts for one-pot reactions^12^,^13^,^14^,^15^,^16^,^17^,^18^. However, general application of these conceptual catalysis in one-pot reactions still needs exploration of highly efficient heterogeneous catalysts with facile and low-cost methods. Science its discovery in 2005^19^, mesoporous carbon nitride (MCN) has aroused extensive interest for its potential applications in organocatalysis^20^, light-driven water splitting^21^, electrochemistry^22^ and CO_2_ capture^23^. Interestingly, this multifunctional material only consists of three fundamental elements (C, H, and N) and can be further functionalized by introducing other elements on its surface for advanced catalysis and nanomaterial applications^20^,^21^,^22^,^23^,^24^. However, creation of bifunctional MCN with acid and base sites is still confronted with great challenge. Typical methods such as H_2_O_2_ or HNO_3_ oxidation to introduce acidic groups into MCN would destroy the intrinsic moderate basic groups of MCN because of over oxidation and thus result in a material with very low even no basicity. Herein, we aim to develop an enzyme-like heterogeneous bifunctional catalyst with acidic and basic functional groups on the surface of MCN through a mild UV irradiation oxidation process which can keep a balance between the inherent basicity and the induced acidity during the oxidation. Owing to the splendid cooperation between acid and base sites, the catalyst shows a very high reactivity and selectivity in a one-pot deacetalization-Knoevenagel reaction.

## Results

### Preparation of the bifunctional MCN (OMCN-1)

**Figure 1:**
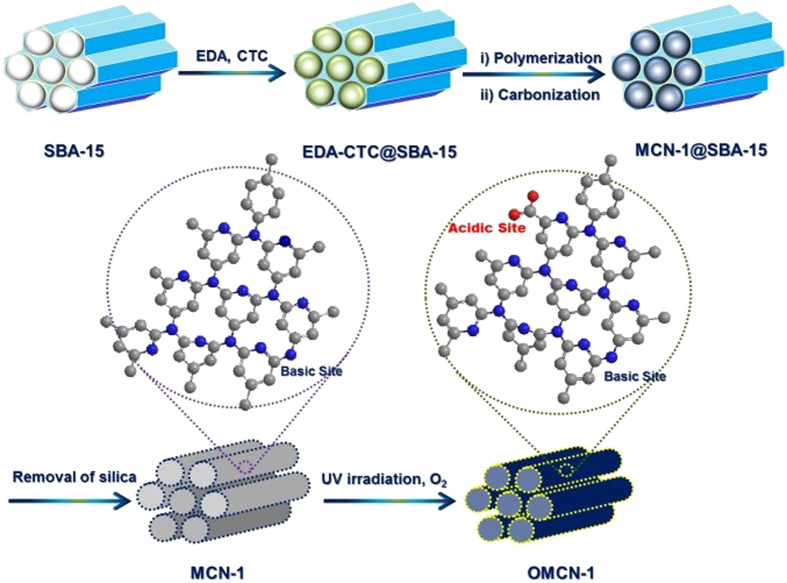
Synthesis of OMCN-1.

The preparation of the bifunctional catalyst is shown in Fig. 1. Briefly, ethylenediamine (EDA) and carbon tetrachloride (CTC) as carbon and nitrogen sources respectively, were first introduced and polymerized in the porous channels of the calcined SBA-15 at 90 °C for 6 h. MCN-1 was obtained by carbonizing the resulting polymer under nitrogen flow followed by removal of the silica template SBA-15 using 5 wt. % HF solution^19^. MCN-1 was then irradiated with UV in oxygen for 2 h to produce the oxidized MCN-1, which is denoted as OMCN-1.

### Characterizations

**Figure 2:**
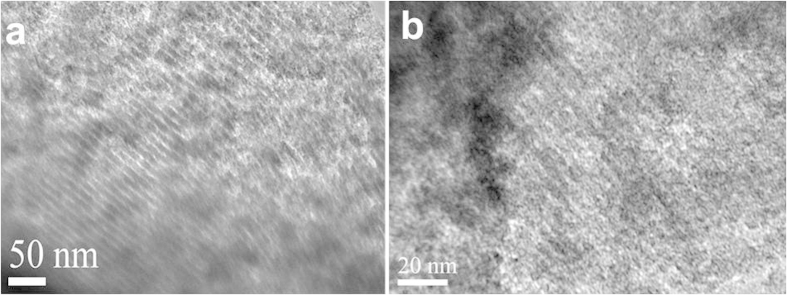
TEM images of MCN-1 (**a**) and OMCN-1 (**b**).

**Figure 3:**
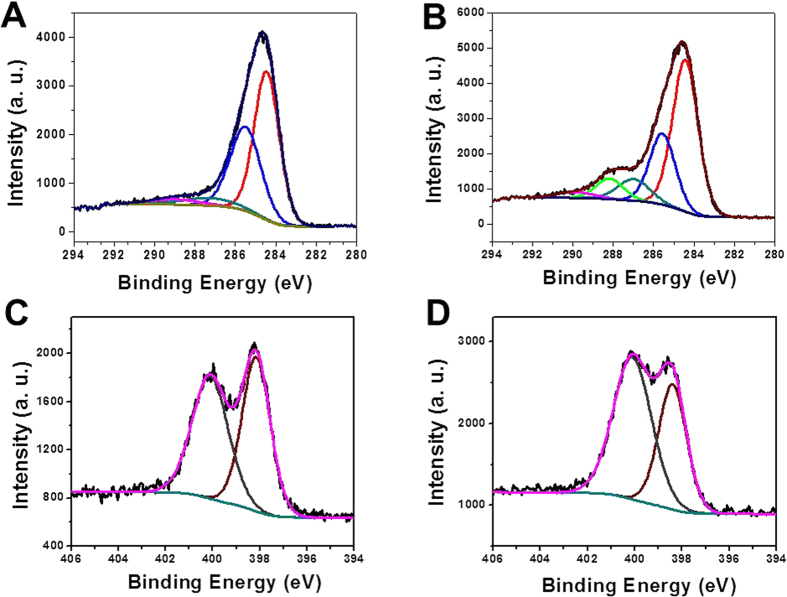
XPS characterizations: (**A**) C1s XPS curve of MCN-1. (**B**) C1s XPS curve of OMCN-1. (**C**) N1s XPS curve of MCN-1. (**D**) C1s XPS curve of OMCN-1.

Creating functional groups on the surface of carbons through oxidation using ozone is considered as a facile and attractive method^25^,^26^,^27^. However, the oxidation process may destroy the structure of the materials to some extent, which is unfavorable for retaining the functionality of the materials. In order to clarify whether MCN-1 could maintain the ordered mesoporous structure replicated from SBA-15 after treatment with UV irradiation in oxygen, MCN-1 and OMCN-1 were characterized by X-ray diffraction (XRD), nitrogen gas adsorption and high resolution transmission electron microscopy (HR-TEM). The XRD patterns of the materials before and after the UV treatment are shown in Fig. S1 (Supplementary Information (SI)). It was found that the XRD pattern for MCN-1 shows two peaks that can be assigned to the (100) and (110) reflection planes which are very similar to those of SBA-15^19^. However, compared with MCN-1, the intensity of the two peaks for OMCN-1 is decreased significantly, which may be due to the marginal loss of structural order. The nitrogen adsorption-desorption isotherm of OMCN-1 is of type IV and also consistent with that of MCN-1. The isotherm of OMCN-1 confirms the mesoporous nature of the material with a pore diameter of 3.8 nm calculated from the desorption branch of the isotherm (Fig. S2, Fig. S3 and Table S1 in SI). The pore diameter and the total pore volume (0.52 cm^3^g^−1^) of OMCN-1 are again comparable to those of the parent MCN-1. However, the specific Brunauer-Emmett-Teller (BET) surface area (400 m^2^g^−1^) of OMCN-1 is decreased by 75 m^2^g^−1^. The decrease may be ascribed to the collapsing of pores during the UV irradiation in oxygen. The TEM characterization was carried out to further investigate the structure of MCN-1 before and after the irradiation. The representative transmission electron microscopy (TEM) images are shown in Fig. 2 where bright contrast strips characterize the pore wall images while dark contrast cores demonstrate the empty channels^19^. The TEM results also confirm the mesostructure and the ordered nature of the pores of OMCN-1. However, as shown in Fig. 2b, the structural order of OMCN-1 reduces slightly and as a result some mesopores in OMCN-1 collapse which is due to the oxidation of the carbon walls by the ozone. The elemental composition of MCN-1 before and after the irradiation was obtained by the CNHO analysis and the results are given in Table S1 in SI. It shows that both MCN-1 and OMCN-1 are mainly composed of carbon and nitrogen with small amount of oxygen, hydrogen and other elements. Oxygen in MCN-1 may come from moisture, ethanol, atmospheric O_2_, or CO_2_ adsorbed on the surface of MCN-1^19^. However, the oxygen content in OMCN-1 is higher than that of MCN-1, which indicates that the groups such as COOH are introduced on the surface of OMCN-1 after the ozone treatment^28^. MCNs are regarded as good adsorbents and both MCN-1 and OMCN-1 can absorb similar oxygen species which come from moisture, ethanol, atmospheric O_2_, or CO_2_^19^. However, after the ozone treatment in which some CH groups of MCN-1 may be oxidized to COOH ones^28^, the oxygen content in OMCN-1 is increased from 8.6 wt% to 10.3 wt%. The generation of the surface COOH groups was confirmed by the Fourier-transform infrared (FTIR) spectrum of OMCN-1. Compared to the FTIR spectrum of MCN-1, the spectrum of OMCN-1 displays a new peak near 1000 cm^−1^ which is attributed to the C-O stretching vibration^29^. In addition, the OMCN-1 spectrum shows a sharper peak at about 3400 cm^−1^ which may be due to the superimposing of the OH stretching vibration to the C-H one (Fig. S4 in SI)^28^. Thus, the two peaks suggest the existence of COOH groups on the surface of OMCN-1. Further characterization for the chemical bonding of the surfaces of MCN-1 and OMCN-1 was conducted by using X-ray photoelectron spectroscopy (XPS) and the results are shown in Fig. 3. It can be seen that the XPS C1s spectra of the two materials have four similar peaks centered at about 284.4 eV, 285.5 eV, 287.2 eV and 289.0 eV, respectively. The four peaks are assigned to pure graphitic sites in the amorphous CN matrix (284.4 eV), the sp2 C atoms bonded to N inside the aromatic structure (285.5 eV), the sp3-hybridized carbon (287.2 eV) and the sp2-hybridized carbon in the aromatic ring attached to NH_2_ groups (289.0 eV), respectively^19^. However, accompanied with the changes of the four peaks in relative area, a new peak (the green profile in Fig. 3B) centered at about 288.2 eV appears in the XPS C1s spectrum of OMCN-1 and this peak could be attributed to carbon atom attached to acidic COOH groups^29^. There are two peaks in the XPS N1s spectra of the two materials: the peak at 398.2 eV assigned to the N atoms trigonally bonded to carbons and the peak at 400.1 eV attributed to the nitrogen sp^2^-bonded to carbon (Fig. 3C,D)^19^,^30^,^31^. Interestingly, the relative area of the peak at 398.2 eV to the peak at 400.1 eV is increased after the irradiation. The increment indicated that some basic groups such as NH_2_ may be oxidized to N-O species^28^. Therefore, the elemental analysis and the XPS measurements clearly demonstrated that the chemical nature, especially acid- base properties of the surface for the two materials are very different due to UV irradiation and the COOH groups are indeed formed on the surface of the OMCN-1.

**Figure 4:**
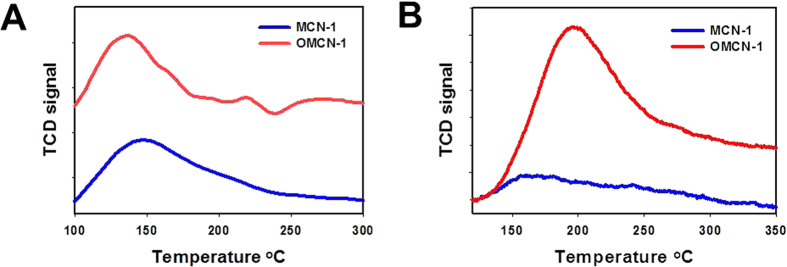
TPD characterizations: (**A**) CO_2_-TPD curves of MCN-1 and OMCN-1. (**B**) NH_3_-TPD-curves of MCN-1 and OMCN-1.

We further investigated the acidic and basic properties of MCN-1 and OMCN-1 using CO_2_-Temperature Programmed Desorption (TPD) and NH_3_-TPD as these two methods are powerful techniques for analyzing the basic and acidic sites on the solid surface, respectively^32^. Figure 4 shows the results of the TPDs. As for MCN-1, there is a peak at 134 °C in the CO_2_-TPD curve and the peak could be assigned to the desorption of CO_2_ which is pre-adsorbed at the weak basic sites on the surface of MCN-1 (Fig. 4A)^33^. The CO_2_ desorption calculated from the area under the peak is about 0.05 mmolg^−1^ and the value is proportional to the amount of the adsorbed CO_2_. Thus, the results indicated clearly that MCN-1 is a material with weak base groups which may be derived from the NH_2_ or NH groups on its surface^24^,^28^,^34^,^35^. Interestingly, a broad NH_3_ desorption peak appeared at about 157 °C in the NH_3_-TPD profile of MCN-1, which means that there are some weak acidic sites on the surface of MCN-1 (Fig. 4A). The calculated desorption of NH_3_ on these acid sites is 0.25 mmolg^−1^. It is expected that the acid sites come from the CO_2_ adsorbed on the surface of MCN-1^19^. Compared with MCN-1, the strength of the CO_2_ desorption peak in the CO_2_-TPD profile of OMCN-1 is weakened and the calculated desorption decreased to 0.03 mmolg^−1^ (Fig. 4B). The decrements may be due to the decline in number of the basic groups such as NH_2_ and NH on the surface of MCN-1 after UV irradiation in O_2_^28^. However, as shown in the NH_3_-TPD curve of OMCN-1, there is a remarkable enhancement of the peak strength of NH_3_ desorption and the peak shifts to a higher temperature of 193 °C with a calculated desorption of 0.75 mmolg^−1^ which is triple the value of the desorption on the MCN-1 (Fig. 4B). These results demonstrate that OMCN-1 is much more acidic than MCN-1 mainly because the oxidation of MCN-1 is significantly facilitated by the UV treatment that could introduce a significant number acidic COOH groups on its surface. Most importantly, about 60% of the inherent basicity of MCN can be still remained after the oxidation, with the result that a concentration “balance” between the remained basic sites and the introduced acidic sites may be established. This balance is very crucial for cooperative performance by the two different groups in acid-base catalytic one-pot transformation.

### Catalytic one-pot reaction

Cooperative catalysis where at least two catalytic sites synergistically activate multiple substrates and increase the rate of the reaction is very common in enzymatic and antibody catalytic processes. For instance, in carbonyl chemistry, acidic and basic sites of enzymes often accomplish catalytic reactions cooperatively with the help of their unique and smart structure^36^,^37^,^38^. Here we try to mimic the structure of enzyme on MCN by introducing both acidic and basic functional group with the simple oxidation using UV treatment. These functionalized enzyme-like catalysts have been used for one-pot reaction that requires both acidic and basic function in order to demonstrate the concept of enzyme-like catalysis (Table 1).

The one-pot reaction involves two steps: deacetalization of benzaldehyde dimethylacetal **1** is first catalyzed by acid sites^39^,^40^, and then in the following Knoevenagel reaction, the resulting benzaldehyde **2** reacts with malononitrile (CH_2_(CN)_2_) to furnish benzylidene malononitrile **3** catalyzed by base sites^41^,^42^,^43^. Surprisingly, the blank reaction showed that the deacetalization can proceed with only a 10% yield of **2** in the absence of any catalyst (Table 1, entry 1). The first reaction step may be catalyzed by small amount of water from solvent. However, the base-catalyzed reaction step cannot occur in the blank reaction which implies the importance of the cooperativity be-tween acid and base sites for the complete one-pot reaction. On the other hand, the pristine MCN-1 showed a low ability to fulfill the one-pot reaction with 50% conversion of **1**, 5% yield of **2** and 45% yield of **3** (Table 1, entry 2). However, to our delight, OMCN-1 exhibited a high efficiency for the one-pot reaction: **1** was converted completely (determined by GC), **3** was obtained with more than 99% yield (GC) and only less than 1% of the final product was **2** (Table 1, entry 3). The immense difference of the catalytic performance between MCN-1 and OMCN-1 may be explained by the fact that OMCN-1 is more acidic than MCN-1 and thus the first reaction step by OMCN-1’s acid groups is kinetically more favorable than that by MCN-1’s acid ones. It is also reasonable that the kinetics in the first step could play an important role in the whole kinetics of the one-pot reaction.

## Discussion

We have carried out only the first step and the second step, respectively, using OMCN-1 as catalyst to further reveal the cooperativity of acid and base sites in kinetics, (Table 1, entries 4 and 5). The results showed that the first reaction was not completed even after 24 h with only 78% yield of **2**. However, the time for converting **2** to **3** with 100% yield (GC) in the second reaction is only 6 h. Therefore, we deduce that the first step of the one-pot reaction is the rate-determining step and the strength of acid sites and the compatibility between acid and base sites are crucial for the cooperative catalysis for the one-pot reaction. When free acid (trifluoromethanesulfonic acid) which is excess to the basic groups on the surface of OMCN-1 is added into the reaction system, the deacetalization take places smoothly while the Knoevenagel reaction doesn’t occur (Table 1, entry 6). However, when the addition is free base (ethylenediamine) which also is excess to the acid sites on the surface, no reaction in both the two steps was observed (Table 1, entry 7). The results confirmed that the confinement of the acidic and basic sites within the nanochannels of MCN that are separated on the nanoporous surface of OMCN-1 is quite important to achieve a high catalytic performance. However, the free groups that are added externally can access these sites and annihilate the opposite groups on the surface thus resulting in destruction of cooperative catalysis and low or even no reactivity for the one-pot reaction.

In summary, we developed and characterized thoroughly a highly efficient bifunctional catalyst OMCN-1 with ordered mesoporous structure through a facile method. The catalyst mainly consists of the simple elements (C, H, O and N) and possesses both acidic sites and basic ones which are distributed separately on the surface of OMCN-1. It was found that the acid-base sites catalyse cooperatively a one-pot deacetalization-Knoevenagel reaction with 100% conversion and more than 99% selectivity. This new enzyme like solid catalyst could provide novel idea and tool for engineering of heterogeneous biomimetic catalyst which could fulfill the objective of simplifying multistep organic synthesis with 100% yields and 100% selectivity.

## Methods

### Synthesis procedure for OMCN-1

4 g of the amphiphilic triblock copolymer was dispersed in water (30 g) and HCl solution (120 mL, 2 M) and stirred for 5 h. Thereafter, tetraethylorthosilicate (TEOS, 9 g)) was added to the homogeneous solution under stirring. The resulting gel was aged at 40 °C for 24 h and finally heated to 100 °C for 24 h. The calcined SBA-15 (0.5 g) was added to a mixture of EDA (1.35 g) and CTC (3 g). The resultant mixture was refluxed and stirred at 90 °C for 6 h. Then, the obtained dark-brown-colored solid mixture was placed in a drying oven for 12 h, and ground into fine powder. The powder was then heat treated in a nitrogen flow of 50 mL per minute at 600 °C with a heating rate of 3.0 °C min^−1^, and kept under these conditions for 5 h to carbonize the polymer. The mesoporous carbon nitrides were recovered after dissolution of the silica framework in 5 wt % hydrofluoric acid, by filtration, washed several times with ethanol and dried at 100 °C to produce MCN-1. Ozonization of the MCN-1 was carried out in an ozone cleaner (Filgen UV253S system, Japan). Oxygen, UV lamp and nitrogen were introduced in order with about 15 minutes, 30 minutes and 5 minutes, respectively. The ozonization was repeated four times resulting in OMCN-1.

### Catalytic one-pot reaction by OMCN-1

Into a Schlenk reaction tube were added OMCN-1 30 mg, benzaldehyde dimethyl acetal (0.5 mmol), CH_2_(CN)_2_ (0.5 mmol), *p*-xylene (as standard) 0.6 mmol, toluene 3 mL. The resulting mixture was stirring under N_2_ at 80 °C. After 24 h, the catalyst was separated by filtration. 10 μl of the filtrate was analyzed by Shimadzu GC-2010 to determine the yields of benzaldehyde and benzylidene malononitrile. The product was purified by chromatography on silica gel and characterized by ^1^H NMR and ^13^C NMR spectroscopy.

### TPD experiments for MCN-1 and OMCN-1

Temperature-programmed desorption of CO_2_ was performed on an AutoChem II 2920. The sample (MCN-1 or OMCN-1) was outgassed at 500 °C under He for 30 minutes then cooled to 100 °C. At the temperature, the sample was saturated with CO_2_ for 1 h. When the baseline reached stable, the sample was heated to 500 °C at a ramping rate of 10 °C min^−1^. The amount of CO_2_ desorbed was monitored by a TCD. Temperature-programmed desorption experiments of ammonia (NH_3_-TPD) were also conducted on the AutoChem II 2920. Before NH_3_-TPD, each sample was pretreated under He at 500 °C for 30 minutes, then saturated with ammonia at 120 °C for 1 h. The sample was then heated to 500 °C at a ramping rate of 10 °C min^−1^. A TCD was used to monitor the amount of NH_3_ desorbed.

## Additional Information

**How to cite this article**: Zhong, L. *et al.* Bifunctional Mesoporous Carbon Nitride: Highly Efficient Enzyme-like Catalyst for One-pot Deacetalization-Knoevenagel Reaction. *Sci. Rep.* **5**, 12901; doi: 10.1038/srep12901 (2015).

## Supplementary Material

Supplementary Information

## Figures and Tables

**Table 1:**
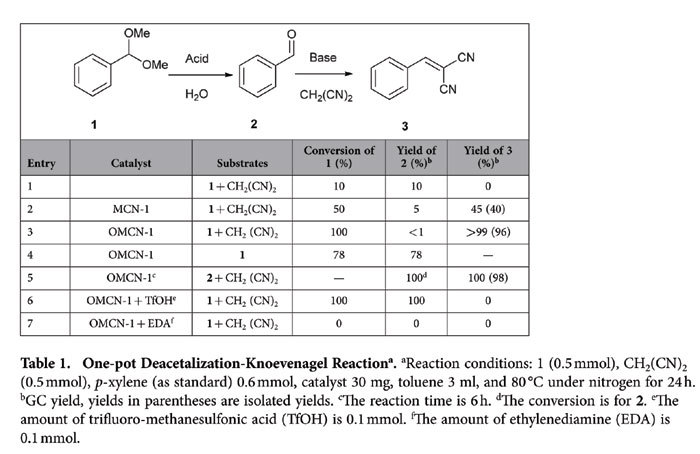
One-pot Deacetalization-Knoevenagel Reaction^a^.
