# Supplementary material for: Bifunctional Mesoporous Carbon Nitride: Highly Efficient Enzyme-like Catalyst for One-pot Deacetalization-Knoevenagel Reaction
Source: Sci Rep. 2015 Aug 5;5:12901. doi: 10.1038/srep12901 (PMC4525375; doi:10.1038/srep12901)

Supplementary Information for

Bifunctional Mesoporous Carbon Nitride: Highly Efficient Enzyme-like Catalyst for One-pot Deacetalization-Knoevenagel Reaction

Lin Zhong[1,2], Anand Chokkalingam[1], Kripal Lakhi[1], Geoffrey Lawrence[1], and Ajayan Vinu[1,*]

[1] *Australian Institute for Bioengineering and Nanotechnology, University of Queensland, Brisbane, Queensland 4072, Australia*

[2] *College of Chemical Engineering, Sichuan University, Chengdu, Sichuan 610065*

Correspondence and requests for materials should be addressed to: a.vinu@uq.edu.au

Materials: Tetraethyl orthosilicate, ethylenediamine (EDA), carbon tetrachloride (CTC), and triblock copolymer poly(ethylene glycol)-block-poly(propylene glycol)-block-poly(ethylene glycol)(Pluronic P123, molecular weight 5800, $EO_{20}PO_{70}EO_{20}$) were obtained from Aldrich. Benzaldehyde dimethyl acetal, malononitrile and *p*-xylene (analytical standard) were also purchased from Aldrich. All the chemicals are commercial available and used as-received.

Characterization: The powder X-ray diffraction (XRD) patterns of the mesoporous materials were collected on a Bruker D8 Advance powder X-ray diffractometer. The diffractograms were recorded in a 2θ range from 0.8 to 10 °with a 2θ step size of 0.01 and a step time of 1 s. Nitrogen adsorption and desorption isotherms were measured at -196 ℃ on a Quantachrome Autosorb 1 sorption analyzer and Micromeritics ASAP 2420. All samples were outgassed at 250 ℃ for 3 h prior to the nitrogen-adsorption measurements. The specific surface area was calculated using the Brunauer-Emmett-Teller (BET) method. The pore size was obtained from the adsorption branch of the nitrogen isotherms using the Barrett-Joyner-Halenda method. Elementary analysis was done using a Flash 2000 Elemental Analyzers. The transmission electron microscopy (TEM) images were obtained using a JEOL 2100 and the accelerating voltage of the electron beam was 200 kV. X-ray photoelectron spectroscopy (XPS)

measurements were carried out in a Kratos Axis Ultra photoelectron spectrometer which useed Al Kα (1253.6eV) x-rays. The FT-IR spectra of the materials were recorded on a Nicolet Nexus 6700 instrument. The ^{1}H NMR and ^{13}C NMR spectra were recorded on a BRUKER 400 spectrometer. The conversion and product yield of the reaction were obtained from Shimadzu GC-2010 with an autosampler. The column is Agilent HP-Ultra 1 (25m * 0.2 mm * 0.33 μm). Split ratio is 50:1, the temperature of vaporization chamber is 270 ℃, and the temperature of FID detector is 270 ℃. The column temperature was controlled by the temperature program: initial temperature is 50 ℃, and the temperature was held for 3 min; then the column was heated with a ramp rate of 10 ℃ / min to 150 ℃ and retained for 3 min; with the same ramp rate, the temperature was raised to 240 ℃ and then reserved for 3 min.

Table S1. Textural parameters and elemental composition for SBA-15, MCN-1 and OMCN-1

Material	A_{BET} [m^2g^{-1}]	V_p [cm^3g^{-1}]	$d_{p, BJH}$ [nm]	C [wt.%]	N [wt.%]	H [wt.%]	O (wt.%)
SBA-15[a]	895	1.20	9.0	-	-	-	-
MCN-1	475	0.56	3.8	63.6	19.4	2.2	8.6
OMCN-1	400	0.52	3.8	57.2	17.7	2.5	10.3

Figure S1. XRD patterns of MCN-1 and OMCN-1 and (inset) HRTEM image of MCN-1.

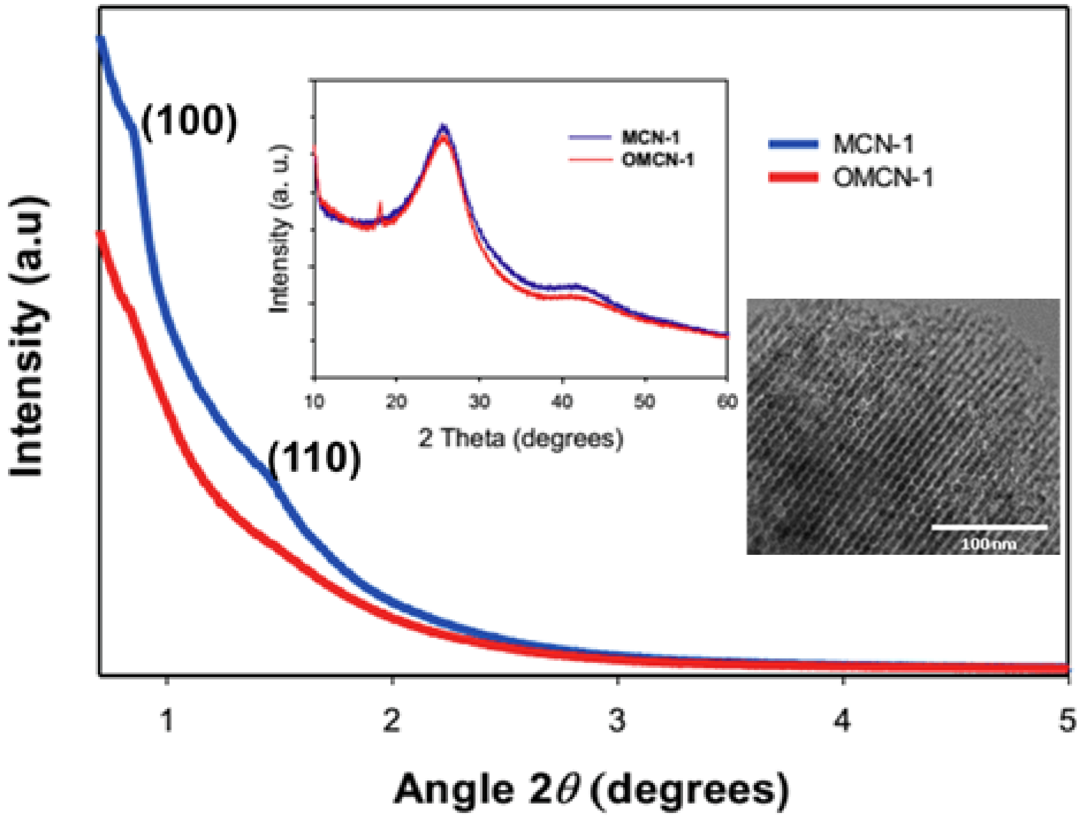

Figure S2A. Nitrogen adsorption isotherm of MCN-1.

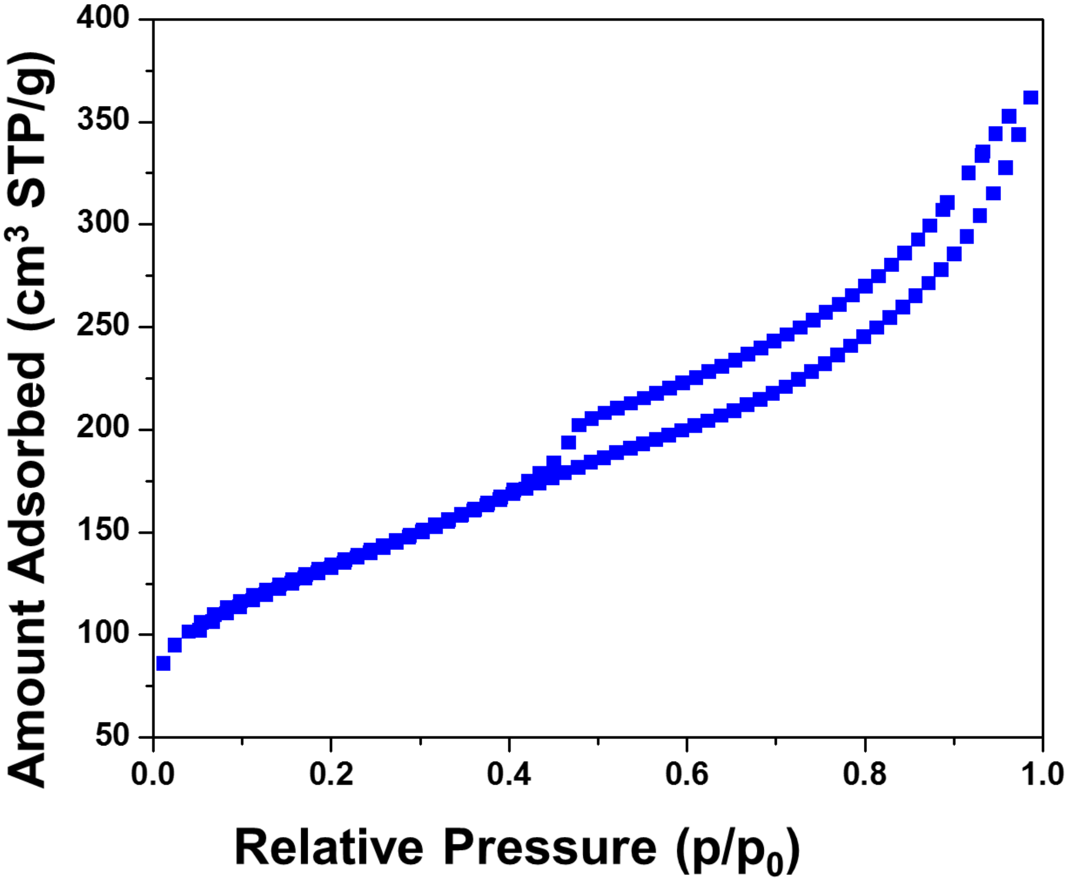

Figure S2B. Nitrogen adsorption isotherm of OMCN-1.

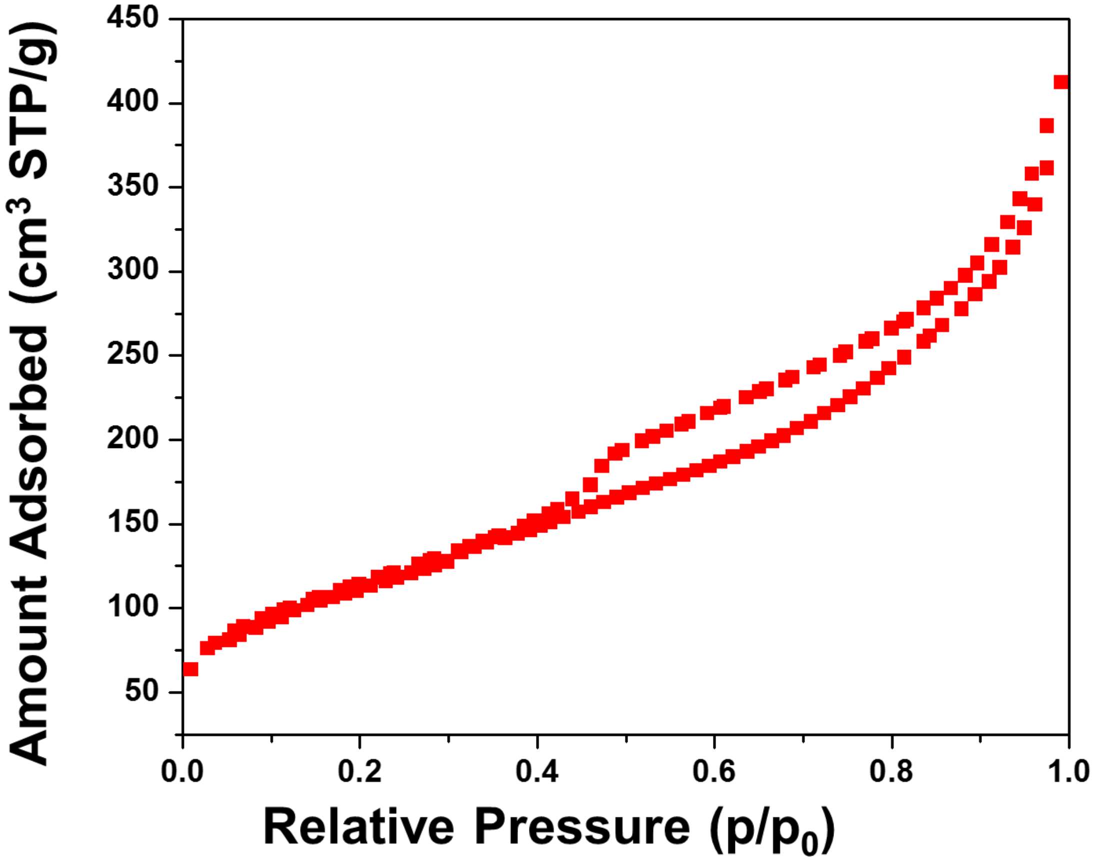

Figure S3. BJH adsorption pore size distribution of MCN-1 (blue) and OMCN-1 (red)

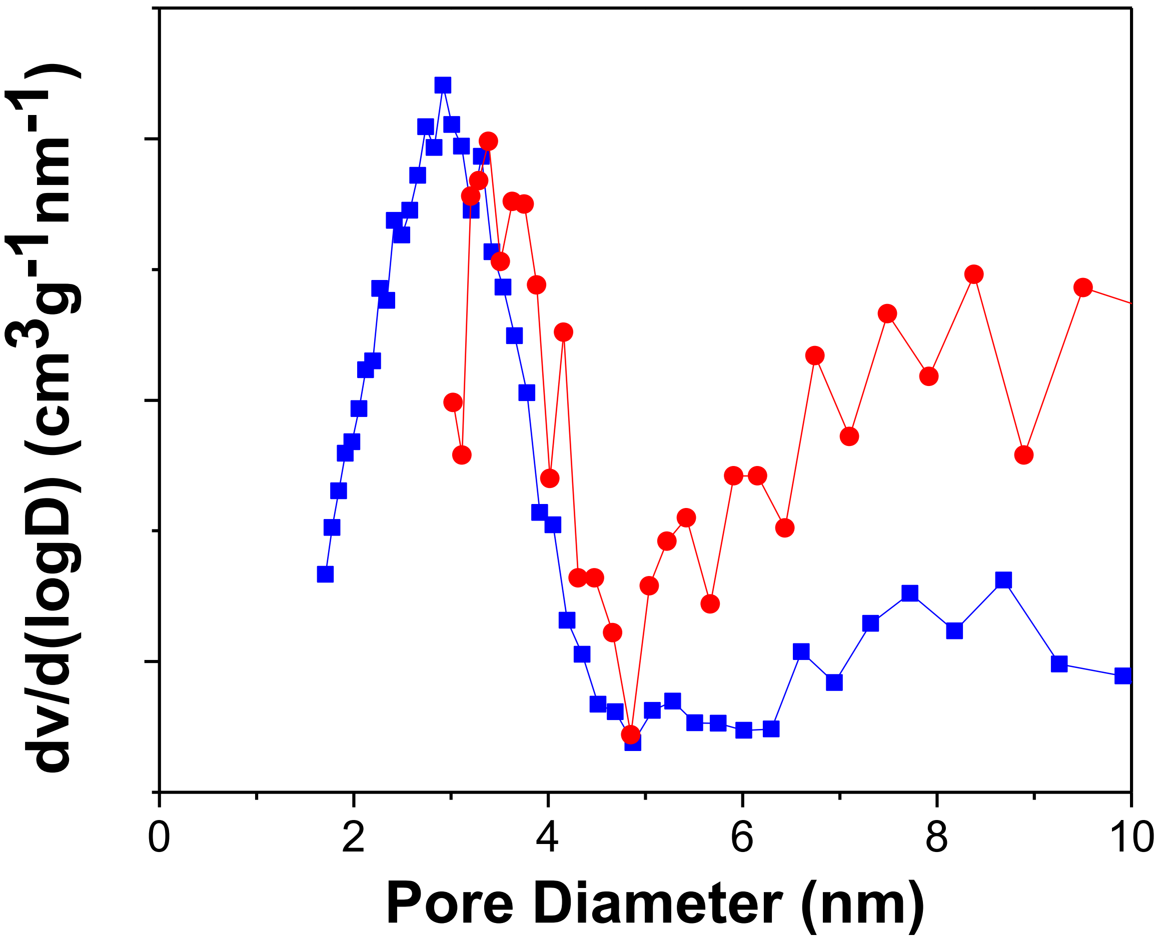

Figure S4. FT-IR spectrum of MCN-1 (blue) and OMCN-1 (red).

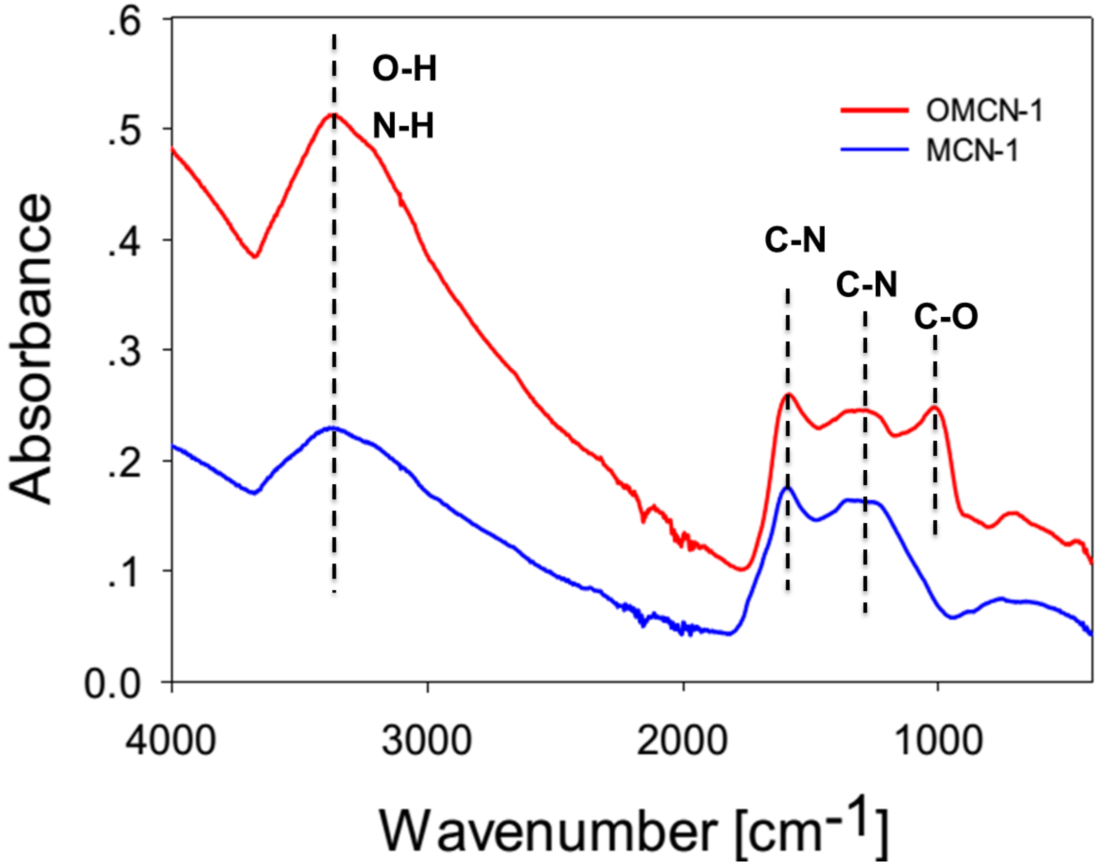

Figure S5. ^{1}H NMR spectrum of the product **3**.

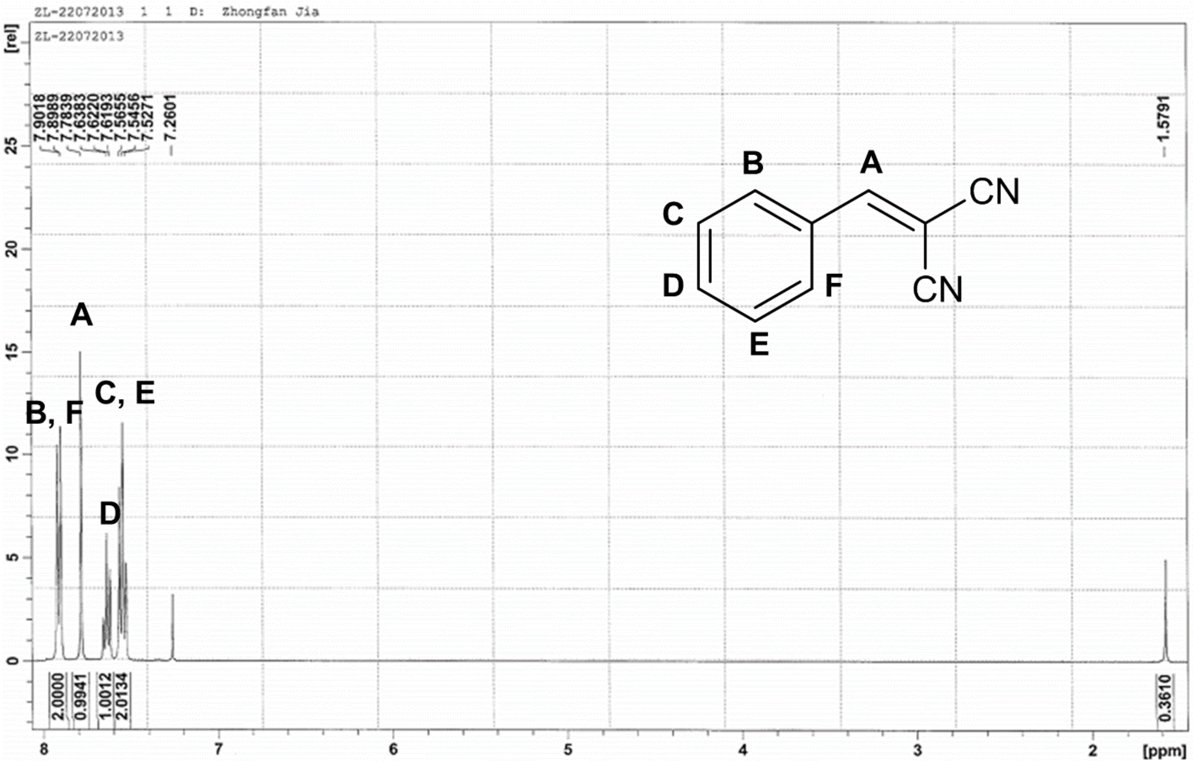

Figure S6. ^{1}H NMR spectrum of the product **3** (Low Field).

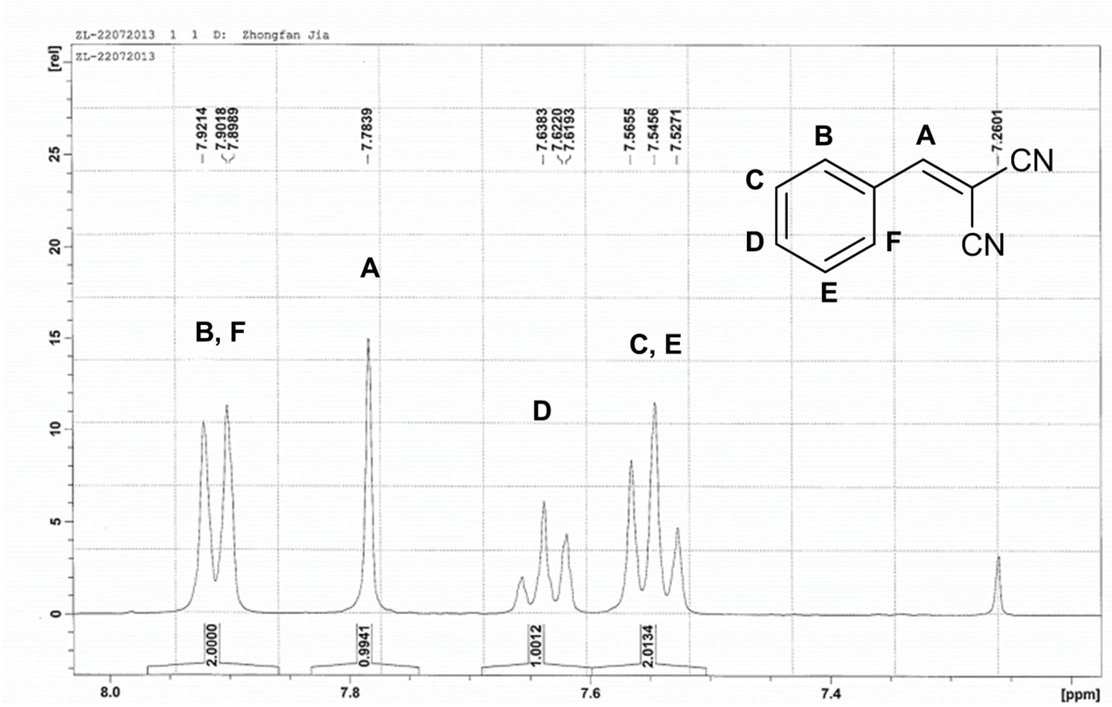

Figure S7. ^{13}C NMR spectrum of the product **3**.

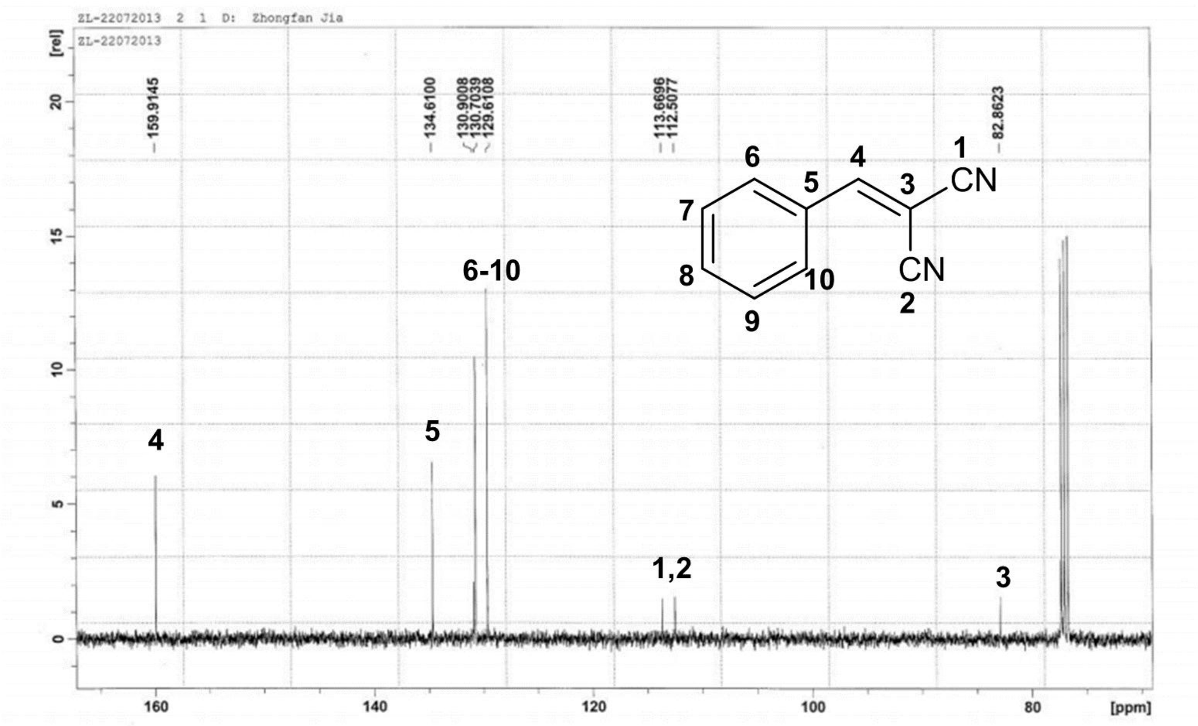

Supplement: Supplementary Information [file srep12901-s1.pdf]
